# Optimising the case-crossover design for use in shared exposure settings

**DOI:** 10.1017/S0950268820000916

**Published:** 2020-05-04

**Authors:** T. Braeye, N. Hens

**Affiliations:** 1 Department of Public Health and Surveillance, Sciensano, Brussels, Belgium; 2 Interuniversity Institute for Biostatistics and Statistical Bioinformatics, Data Science Institute, Hasselt University, Hasselt, Belgium; 3 Centre for Health Economics Research & Modelling Infectious Diseases (CHERMID), Vaccine & Infectious Disease Institute, University of Antwerp, Antwerp, Belgium

**Keywords:** Case-crossover study, conditional logistic regression, simulation study

## Abstract

With a case-crossover design, a case's exposure during a risk period is compared to the case's exposures at referent periods. The selection of referents for this self-controlled design is determined by the referent selection strategy (RSS). Previous research mainly focused on systematic bias associated with the RSS. We additionally focused on how RSS determines the number of referents per risk, sensitivity to overdispersion and time-varying confounding.

We illustrated the consequences of different RSS using a simulation study informed by data on meteorological variables and Legionnaires’ disease. By randomising the events and exposure time series, we explored statistical power associated with time-stratified and fixed bidirectional RSS and their susceptibility to systematic bias and confounding bias. In addition, we investigated how a high number of events on the same date (e.g. outbreaks) affected coefficient estimation. As illustrated by our work, referent selection alone can be insufficient to control for a time-varying confounding bias. In contrast to systematic bias, confounding bias can be hard to detect. We studied potential solutions: varying the model parameters and link-function, outlier-removal and aggregating the input-data over smaller areas. Our simulation study offers a framework for researchers looking to detect and to avoid bias in case-crossover studies.

## Introduction

The case-crossover method is an efficient study design for investigating associations between transient exposures during a risk period and the onset of acute events [1]. The design is self-controlled and therefore it is free from time-invariant confounding and does not require the inclusion of unaffected controls. It does, however, require the inclusion of one or multiple time periods to serve as referents to the risk period. The risk period typically precedes the event by a number of days. The selection of referents was a topic of methodological development and discussion for several years after the case-crossover study design was originally introduced by Maclure in 1991 [2]. This is best illustrated by how recommendations for the selection of referents have evolved from, using the taxonomy developed by Janes *et al*. [3], fixed unidirectional to bidirectional to time-stratified referent selection [4]. To understand this evolution, we need to introduce the concept of exchangeability and time-varying confounding [5].

Exchangeability originated from Bayesian statistics indicates that sequences of random variables have the same joint probability distribution [6]. Consider the simple example in which an exposure time series has a linear upward trend. A referent selection strategy (RSS) that samples referents prior to the risk period, known as a unidirectional RSS, will always detect lower exposure values on referent dates as opposed to on risk dates. Exposure values on risk dates will be non-exchangeable with exposure values on referent dates [5]. Navidi, therefore, proposed bidirectional or ambidirectional sampling. With these strategies, referents are selected both before and after the risk period [7]. Levy *et al*. established that even when exposure after the event is irrelevant (e.g. because the event was death) bidirectional sampling is preferred to unidirectional because the bias in coefficient estimation will be smaller [8]. Referents were selected from fixed time periods close to the risk period to limit time-varying confounding. Referents should preferably not be selected adjacent to the risk period to avoid possible carry-over effects. Bateson and Schwartz continued the development of RSS by pointing to a selection bias associated with fixed bidirectional sampling [9, 10]. Eventually, the dates at the beginning and end of the period under investigation will be selectable only as referents. If we select the last date in the period under investigation as a risk, a referent can only be selected prior to the risk. This results in unidirectional referent selection. Time-stratified referent selection was proposed by Janes *et al*. as a final solution to avoid the bias associated with non-exchangeability [3]. They named this bias ‘overlap bias’ in accordance with the terms used in matched case−control studies. Their solution consists of dividing the period under investigation in strata (smaller time periods). All dates within a stratum are used as referents for the risk date in the same stratum. While time-stratified RSS are free from overlap bias, there are many ways to divide a time period into strata. There is no single optimal RSS for all datasets. Researchers have to keep time-varying confounding and statistical power in mind when deciding which time-stratified RSS to use.

Once referents are selected, exposure values on both risk and referent dates are fitted to a model. The conditional selection of referent dates on risk dates should be reflected in the model. To achieve this, case-crossover studies typically use conditional logistic regression models. Conditional Poisson models have been suggested as flexible alternatives by Armstrong *et al*. [11]. They advocated the use of conditional Poisson models because these models can be adjusted for overdispersion and autocorrelation. Noteworthy is that the equivalence between case-crossover conditional logistic models and time series conditional Poisson models with stratum indicators in environmental epidemiology has been established [12].

We investigated short-term associations between meteorology and Legionnaires’ disease incidence as an illustrative example. We used simulated data informed by Belgian data on meteorological variables and Legionnaires’ disease cases. Several researchers have already applied case-crossover designs in studies on Legionnaires' disease incidence and meteorological variables. The RSS they have used varied from restricted unidirectional RSS, selecting referents 1 and 2 weeks before the risk [13], to several time-stratified RSS. These time-stratified RSS included: strata of 21 days with referents selected at the same day of the week [14–16], strata of 28 days with referents selected as all other days [17] and ‘day of the year’-strata in which corresponding days from the other years of the study period were selected as referents [18].

Research on the association between Legionnaires' disease-incidence and meteorological variables also presents additional problems: unknown individual exposures and overdispersion. The problem of unknown individual exposures is common in epidemiological and environmental research [19]. The problem might be more complex in an infectious disease context. In addition to a person's exposure, exposure at the source and place of infection are also relevant. Consequently, convenient approaches, such as setting the exposure values for large areas equal to exposures measured at local monitoring stations, appear inevitable. This is not necessarily problematic. Whenever the average of the actual exposures equals the value used in the analysis, the measurement error is considered a Berkson-error and the analysis is expected to yield an unbiased effect estimate, provided that the true dose-response is linear [20]. While it is established that Berkson-error reduces power, it is hard to estimate how much power is lost if we would aggregate over a single large area as opposed to aggregating over multiple smaller areas [21]. A second issue with infectious disease data is overdispersion. Legionnaires' disease is likely driven by unknown variables or variables that cannot be included in the model. Multiple events might be linked to unidentified outbreaks. These outbreaks will create extra variability, overdispersion, in the events time series. An analysis with a conditional logistic model might not be appropriate when overdispersion is present.

While systematic bias has been the focus of previous research, there are other important consequences to different RSS. We investigated confounding bias, overdispersion and statistical power associated with different RSS in a simulation study. We extended our illustrative example by also considering the effects of data preparation, particularly the size of the area over which to aggregate and model fitting, particularly conditional Poisson models *vs.* conditional logistic models.

## Methods

### Data on legionnaires' disease and meteorological variables

The Legionnaires' disease case data included in the simulation study was obtained from three different surveillance sources: the national reference centre, the laboratory sentinel surveillance network and regional mandatory notification [22, 23]. Additional information on the data sources can be found in the attachment. Since electronic records for Legionnaires' disease were available from 2004 and data analysis for this paper started in 2018, the study period was set from 2004 to 2017. Data sources were combined and duplicates were identified. Duplicated were defined as having the same sex, birthdate and postal code and dates of diagnosis that were within a 30 days period. The latest recorded duplicates were removed. Case definitions can be found in the attachment. To include as many cases as possible and as the date of onset was missing for most cases, the date of diagnosis was used as the event date.

We included daily values for three meteorological variables in the simulation study (temperature (°C), relative humidity (%) and wind speed (metre/second)). The data were obtained from the Royal Meteorological Institute of Belgium for all available weather stations that recorded data from 2004 to 2017 (*N* = 29). We applied a linear transformation (standardisation) that rescaled the three meteorological factors. For meteorological variable (*x*), this was calculated as:

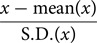

The data were prepared in two different ways: (1) the national analysis in which we aggregated all data by date and (2) the provincial analysis in which we aggregated the data by province and date.

### Simulation scenarios

We altered the data for analysis using three different simulation scenarios:

1. ‘Random events, unaltered exposures': in the first scenario we created 2277 events. These events were given a random date between 1 January 2004 and 31 December 2017. We left the exposure time series unaltered. As the event dates were selected randomly, the probability of an event was not determined by exposure values and confounding was not possible. Whenever coefficients were estimated at a value different from zero, a systematic bias was present.
2. ‘Unaltered events, random exposures': in the second scenario, we left the events time series unaltered and randomly allocated exposure values to dates. With this scenario, we assessed non-exchangeability within matched sets of risks and referents and the influence of overdispersion. Overdispersion manifested when a high number of events occurred on the same date. We defined outlier-dates as dates on which five events or more occurred. We investigated overdispersion in two sub-scenarios: one in which events on outlier-dates were removed and one in which they were not.
3. ‘Event probability modelled by exposure values': in the third scenario, the occurrence of events was determined by the exposure values 6 days earlier, a seasonal trend and a long-term trend. The exposure time series was unaltered. With this scenario we explored if confounding between the seasonal and long-term trends and the effect of the exposure values remained present after referent selection. Time-varying confounding was present if the coefficients estimated by the case-crossover model differed from the coefficients used during the creation of events. The model for estimating the event probabilities was:

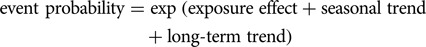

with

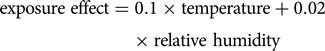


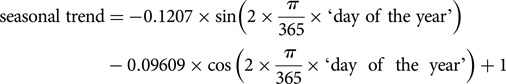


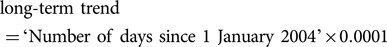

**Fig. 1.**
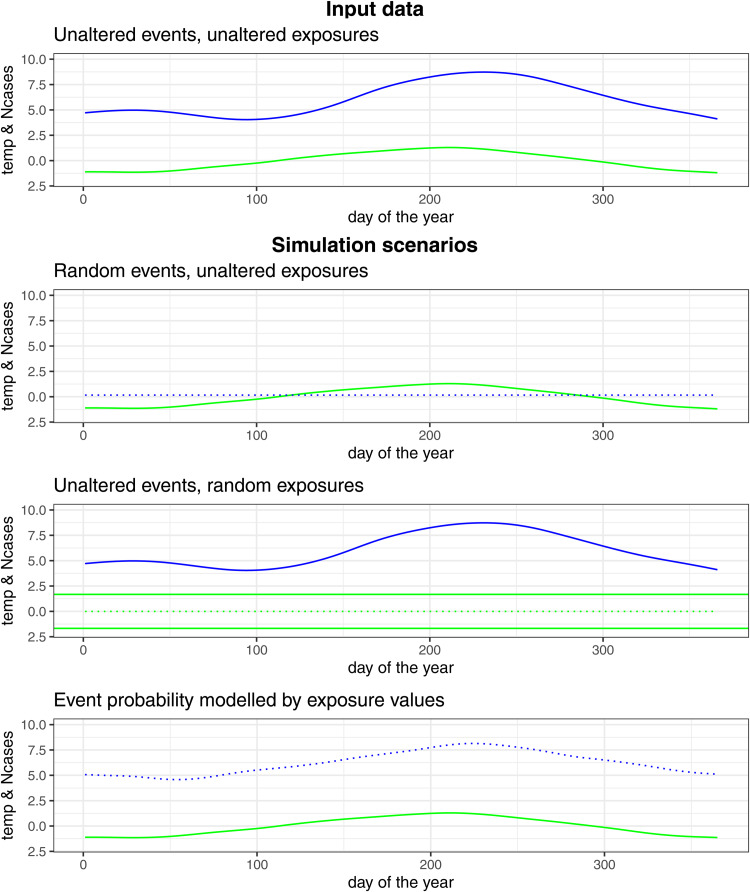
Graphical representation of the events and exposures time series in the different simulation scenarios (green = temperature, blue = number of cases (Ncases), dotted line = random, full line = unaltered).

A graphical representation of the three simulation scenarios is given in Figure 1. In all scenarios, we worked with 2277 event dates over a time period from 2004 to the end of 2017. Each simulation scenario was repeated 5000 times. We presented the obtained coefficients in boxplots and we presented line plots in which the number of significant coefficients is plotted over the nominal (advertised) type I error or the risk period.

### Referent selection

**Fig. 2.**
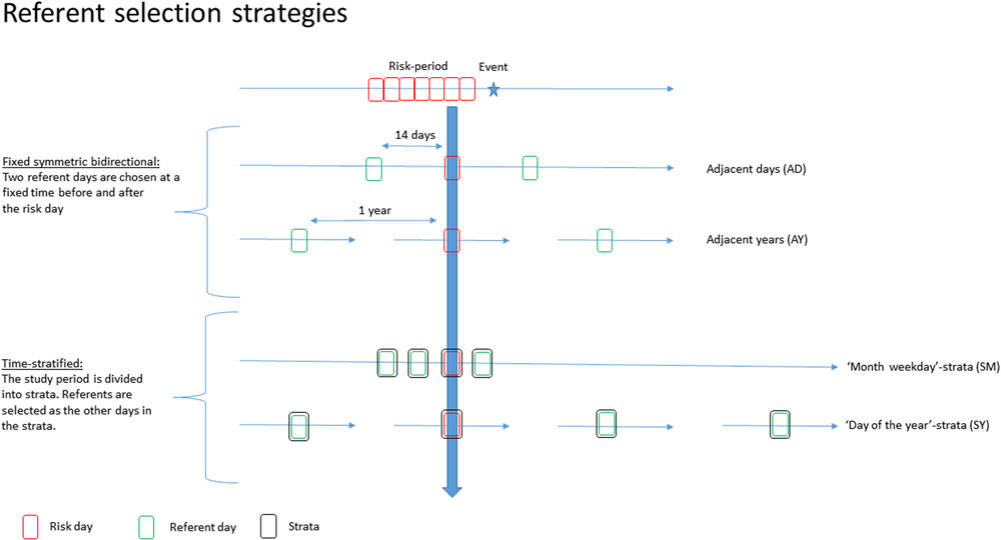
Overview of the different referent selection strategies.

We explored four different RSS; two fixed bidirectional RSS; adjacent days (AD), adjacent years (AY) and two time-stratified RSS; strata month-weekday (SM) and strata day-of-the-year (SY). With the fixed bidirectional RSS, referents were selected at a fixed period before and after the risk. These fixed periods were 14 days (AD) and 1 year (AY). With the time-stratified RSS, the study period was divided into strata. These strata could differ in length and did not have to be consecutive. With the SM RSS, strata consisted of all the days during a certain month and a certain year that were the same day of the week (e.g. all Mondays from January 2012). With the SY RSS, strata consisted of all the days that occurred on the same day of the year (e.g. all the first days of the year) (Fig. 2).

### Data analysis

We fitted exposure values at the risk day and exposure values at the referent days with a conditional regression model. For the ‘random events, unaltered exposures’-scenario we fitted a conditional logistic model to each of the datasets. This resulted in 40 000 models (5000 simulations × 4 different RSS × 2 Province/National analysis). In the ‘unaltered events, random exposures’-scenario, we again fitted conditional logistic models to each of the datasets (40 000 models). In addition, two conditional quasi-Poisson models were fitted to the SM- and SY-data (20 000 models = 5000 simulations × 2 RSS (SM/SY) × 2 Province/National analysis). We named these SM.cp and SY.cp. We refitted all the models from the second scenario after the removal of the outlier-dates and referents from the input-data (60 000 models). In the ‘event probability modelled by exposure values’-scenario, we fitted the data from the AY and AD RSS with conditional logistic models, the SM and SY RSS were fitted with conditional quasi-Poisson models (SM.cp/SY.cp). We fitted two additional models to which additional time-varying variables were added (SM.m.cp/SY.m.cp) (60 000 models per risk day). The additional time-varying variable for SM.m.cp was a sinusoid 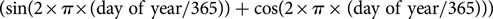 and the additional time-varying variable for SY.m.cp was the number of years since the start of the study period (Table 1).

**Table 1.** Overview of the different models. The name refers to the referent selection and the model used for fitting the data after referent selection

Name	Formula	Conditioning on
Conditional logistic regression model		
AD, AY, SM, SY	logit(Probability of event) ~ temperature + rel. humidity + wind-speed	Unique event identifier
Conditional quasi-Poisson regression model		
SM.cp, SY.cp	log(Number of events) ~ temperature + rel. humidity + wind-speed	Strata
SM.m.cp	log(Number of events) ~ temperature + rel. humidity + wind-speed + sinusoid	
SY.m.cp	log(Number of events) ~ temperature + rel. humidity + wind-speed + year	

AD, adjacent days; AY, adjacent years; SM, strata month-weekday; SY, strata day-of-the-year; m, additional time-varying variable; cp, conditional Poisson.

The ‘sinusoid’ is defined as: sin(2 × *π* × (doy/365)) + cos(2 × π × (doy/365)) and the ‘year’ is the number of years since the start of the study period (2004).

### Software and code

All analyses were performed in R. The gnm-package was used for conditional Poisson analysis [24]. All code was made available on https://zenodo.org/badge/latestdoi/245381376.

## Results

### Simulation results

#### Random events, unaltered exposures

**Fig. 3.**
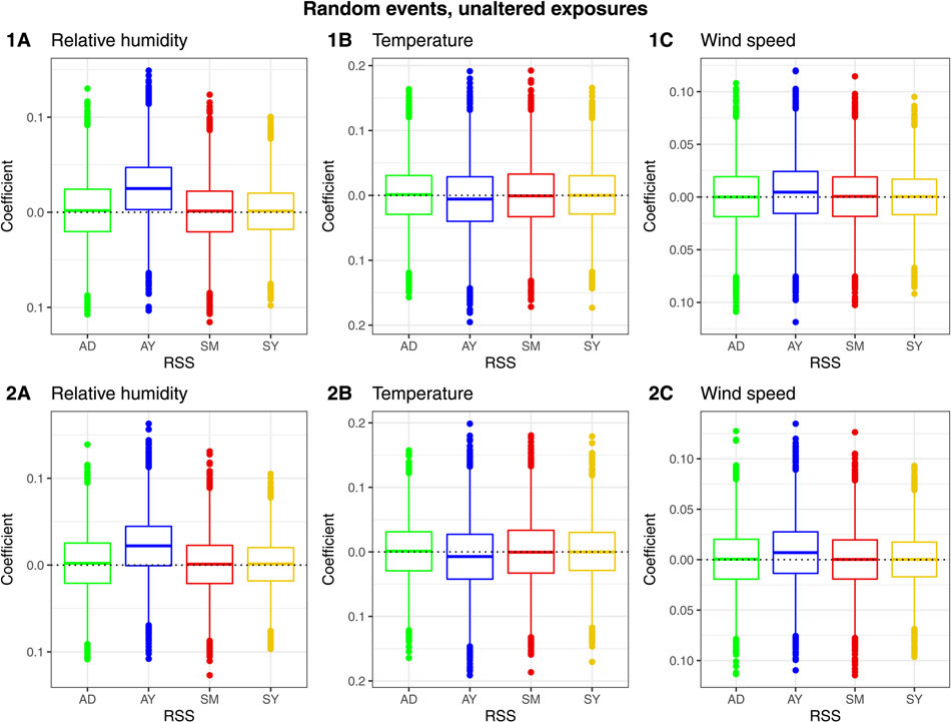
Scenario ‘random events, unaltered exposures’. Boxplots of the coefficient estimates per exposure (relative humidity (A), temperature (B), wind speed (C)) and per RSS (AD = adjacent days, AY = adjacent years, SM = strata month-weekday, SY = strata day-of-the-year). Aggregation on (1) national (2) provincial level.

*Coefficient estimation.* The time-stratified designs (SM, SY) resulted in visually unbiased coefficients. The systematic bias associated with AD was small for relative humidity and temperature and visually absent for wind speed. The systematic bias associated with AY was present for all variables. The biases associated with AY and AD remained present in the provincial analysis (Fig. 3).

**Fig. 4.**
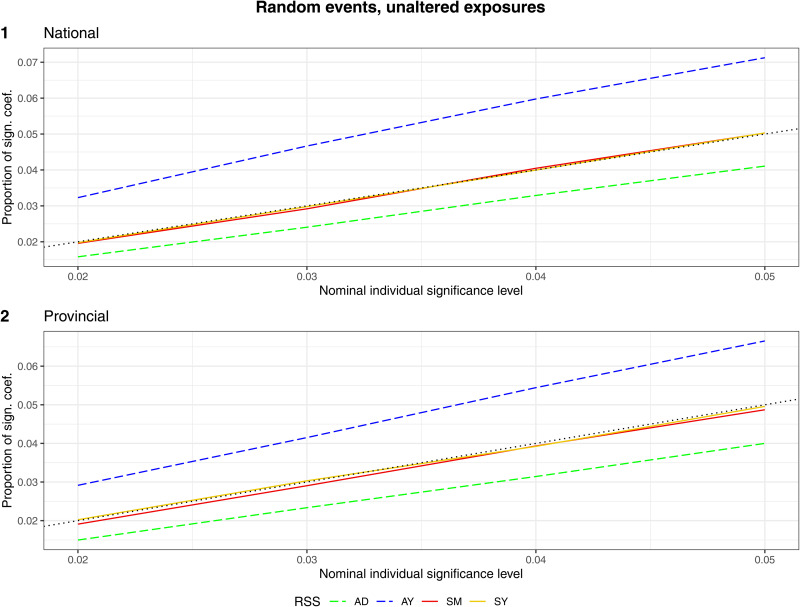
Scenario ‘random events, unaltered exposures’. The proportion of significant coefficients over the nominal individual significance level by RSS (AD = adjacent days, AY = adjacent years, SM = strata month-weekday, SY = strata day-of-the-year). Aggregation on (1) national (2) provincial level.

*Proportion of significant coefficients.* The time-stratified RSS (SM, SY) resulted in a proportion of significant coefficients close to the nominal significance level. The other RSS either resulted in a higher (AY) or lower (AD) proportion of significant coefficients. When the data were aggregated on a provincial level instead of on a national level, this slightly decreased the proportion of significant coefficients for AY (Fig. 4).

#### Unaltered events, random exposures

**Fig. 5.**
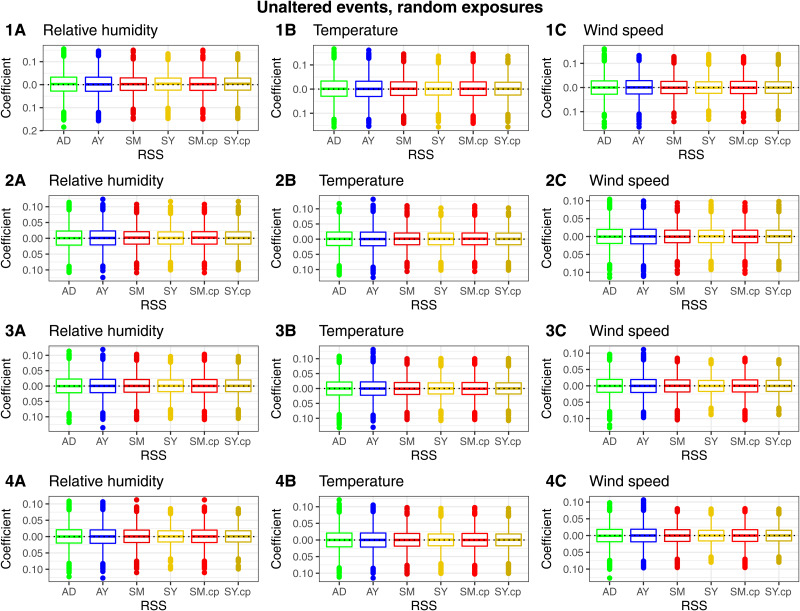
Scenario ‘unaltered events, random exposures’. (1) Boxplots of the coefficient estimates per exposure (relative humidity (A), temperature (B), wind speed (C)) and per RSS (AD = adjacent days, AY = adjacent years, SM = strata month-weekday, SY = strata day-of-the-year, SM.cp = strata month-weekday quasi-Poisson, SY.cp = strata day-of-the-year quasi-Poisson). Aggregation on (1) national, (2) national no-outliers, (3) provincial, (4) provincial no-outliers level.

*Coefficient estimation.* Coefficient estimation was unbiased for all RSS. The coefficients associated with the time-stratified SM and SY fitted with a conditional logistic model (SY and SM) were equal to those fitted with a conditional quasi-Poisson model (SY.cp and SM.cp) (Fig. 5).

**Fig. 6.**
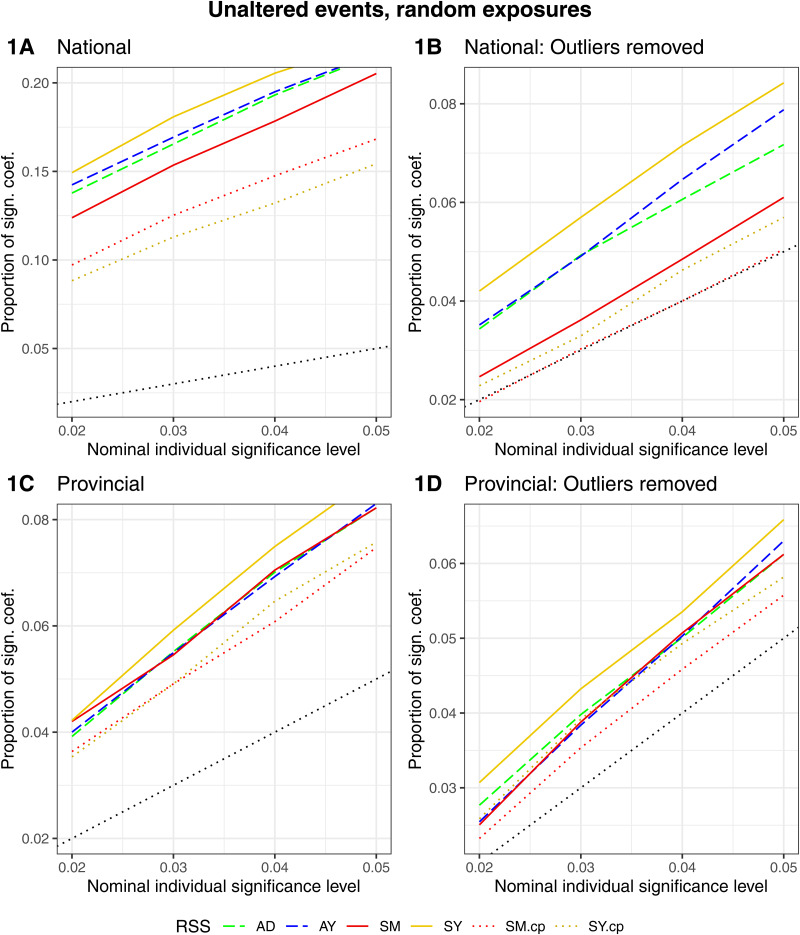
Scenario ‘unaltered events, random exposures’. The proportion of significant coefficients over the nominal individual significance level by RSS (AD = adjacent days, AY = adjacent years, SM = strata month-weekday, SY = strata day-of-the-year, SM.cp = strata month-weekday quasi-Poisson, SY.cp = strata day-of-the-year quasi-Poisson). (1A) National, (1B) national no-outliers, (1C) provincial (1D) provincial no-outliers.

*Proportion of significant coefficients.* We obtained a proportion of significant coefficients that was higher than the nominal level with all RSS in all four analyses (no-outlier/outlier-dates and province/national analysis). This indicated that trends remained present in the matched sets of risks and referents. These trends were partly eliminated by removing the strata with outlier-dates and during provincial analysis. The Removal of strata with outlier-dates resulted in the largest reduction of the proportion of significant coefficients. The four outlier-dates accounted for a total of 76 events. As for differences between RSS: AY and AD resulted in a comparable number of significant coefficients. For the time-stratified RSS: SY resulted in more significant coefficients compared to SM for the national-level analysis, the difference was smaller when data were aggregated by province. The quasi-Poisson models (SM.cp and SY.cp) brought the proportion of significant coefficients closer to the nominal level in all analyses (Fig. 6).

#### Event probability modelled by exposure values

*Coefficient estimation.* AY provided biased estimates for all three variables. Due to confounding, the time-stratified RSS (SM and SY) provided estimates with a larger bias than AD. The time trends remaining within the matched sets confounded with the exposure effects. For example, there was positive confounding between the temperature effect and seasonality with SM and negative confounding between the wind speed-effect and SY. The confounding bias was slightly smaller in the provincial analysis.

The RSS with additional within strata modelling (SM.m.cp and SY.m.cp) provided unbiased estimates.

*Proportion of significant coefficients.* The SY allowed for more statistical power as compared to the SM. This was especially true for the estimation of the temperature effect. The inclusion of extra variables to model time trends and eliminate time-varying confounding within SY.m.cp and SM.m.cp lowered power. With these models, we found that relative humidity was the only variable for which the national analysis was less powerful than the provincial analysis. The Berkson-error was small.

**Fig. 7.**
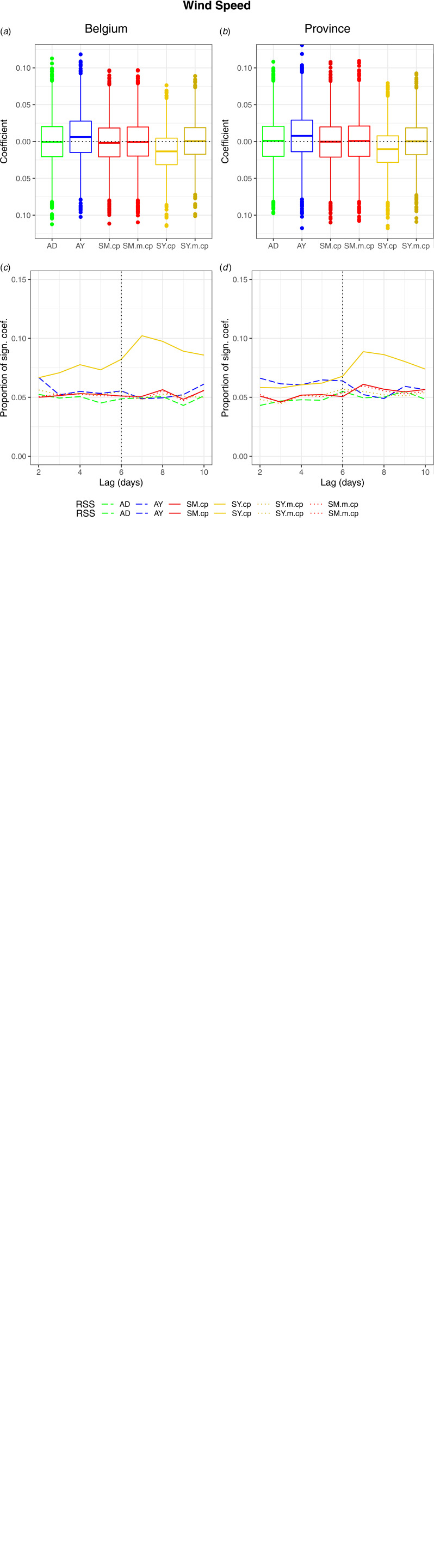
Scenario ‘event probability modelled by exposure values’. (A&B) Boxplots of the coefficient estimates by RSS. (C & D) The proportion of significant coefficients over the nominal individual significance level by RSS. (A & C) National. (B&D) Provincial. (AD = adjacent days, AY = adjacent years, SM = strata month-weekday, SY = strata day-of-the-year, SM.m.cp = strata month-weekday quasi-Poisson model with an additional variable for seasonality, SY.m.cp = strata day-of-the-year quasi-Poisson model with additional time-varying variable).

While the exposure effect concerned the 6th day prior to the event, autocorrelation within the exposure series resulted in significant coefficients for the exposures on risk days around the 6th day. All RSS were affected (Fig. 7).

## Discussion

Whitaker *et al*. discouraged the use of case-crossover-methods: ‘these methods are either biased, or are special cases of more versatile methods’ such as time series regression methods [4]. The case-crossover design, however, remains a popular design. Its popularity is likely caused by its familiarity and apparent simplicity; it does not require explicitly modelling seasonality or other time-varying trends. In addition, it typically allows for fast model fitting as it is a conditional analysis and does not require coefficients of time-varying trends to be estimated. Knowing both the popularity of the design as well as the challenges with referent selection, we tried to set up a case-crossover study that allowed for unbiased coefficient estimation and was versatile enough to model our data.

### Bias

#### Systematic and confounding bias

The underlying mechanism of confounding bias and systematic bias in case-crossover studies is the same: within a matched set of risk- and referent-dates some dates are more likely risk than referent and these unequal probabilities coincide with trends in the exposure series. All our three simulation scenarios explored this mechanism. In the ‘unaltered events, random exposures’-scenario there were no trends in the exposure series and therefore there was no bias. In the absence of trends in the exposure series, the assumption of global exchangeability is always valid. Global exchangeability is defined as the independence of outcome probabilities conditional on the causal effect of exposure across all dates within a matched set [25, 26]. Whenever there were trends in the exposure time series (non-exchangeability), the fixed bidirectional RSS inherently resulted in bias. Even if trends in the events time series were absent as in the ‘random events, unaltered exposures’-scenario, a bias was present and this, therefore, is considered a systematic bias. Once trends were present in the events time series, as in the ‘event probability modelled by exposure values’-scenario, confounding bias occurred with all RSS. For fixed bidirectional RSS, a confounding bias cannot be separated from a systematic bias. The confounding bias associated with the time-stratified RSS is not systematic as it is a function of the referent selection, the event time series and the exposure time series.

#### The size of the bias

The type of bias did not determine its size. The systematic bias associated with fixed bidirectional RSS is determined by the proportion of affected matched sets and the trends in the exposure time series. It will be minimal if the exposure time series is relatively stationary and whenever the ‘true’ relative risk is close to one [27]. The proportion of affected matched sets was small for AD (the last and first 14 days of a 13-year study period) and large for AY (the first and last year of the study period). This resulted in higher biases for AY. Researchers have suggested to avoid overlap bias by not including events from either end of the study period [28]. This can increase bias however as the problem persists (for example for AY: if events from 2004 are removed, the events from 2005 are now part of matched sets in which referents selected before the event cannot be risks) and the proportion of affected matched sets has risen. In contrast to a systematic bias, a confounding bias is harder to detect and its size is harder to predict. Vulnerability to confounding is determined by several factors. For example, except for the matched sets at both ends of the study period, fixed bidirectional RSS are not vulnerable to confounding with linear long-term trends, while time-stratified RSS are. This is caused by the asymmetry within matched sets of time-stratified RSS: there is one date in each stratum for which all referents are later dates. As presented in the ‘events probabilities modelled by exposure values’-scenario, the (systematic) bias of AD was smaller than the bias associated with time-stratified RSS. While this finding is specific to our study, it is interesting to note that a fixed bidirectional RSS provided the least biased estimates, in spite of two time-stratified RSS also included in the study.

While the systematic bias associated with unidirectional and fixed bidirectional RSS is insoluble, there are three solutions to confounding bias. All three solutions have limitations. First, we can further modify our time-stratified referent selection. The seasonality confounding found in SM could be limited by making the strata shorter (e.g. 3-week strata instead of 1-month strata, a strategy taken by [14–16] and explored in the Supplementary files). Shorter strata will, however, lead to fewer referents per risk and the autocorrelation between referents will further limit the efficiency of such an RSS. Modelling within strata is a second possible solution. This however also has several limitations; e.g. a year-effect necessarily is equal over SY-strata and the inclusion of extra variables limits power. Third, we had less confounding while aggregating the data on a provincial instead of a national level. This is specific to our dataset, e.g. in some of the Belgian provinces, there was no long-term trend in the event series. In general, it is however likely that different seasonality and long-term trends can be found within more and smaller areas.

### Variance and power

In addition to the bias associated with coefficient estimation, we also presented the proportion of significant coefficients for our different simulation scenarios. When the assumption of exchangeability is violated, the assumption of independent events essential for maximum likelihood estimation of the conditional logistic regression model is also violated. A direct consequence is that the Poisson variance associated with the number of events per day is insufficient [29]. We explored this with two quasi-Poisson models in the ‘unaltered events, random exposures’-scenario. The overdispersion in these models measured the unmodelled unequal probabilities (within matched sets for a date to be selected as a risk-date). This was larger for SY than for SM. Confounding can lead to erroneously estimated coefficients and misguided confidence, represented by a large proportion of significant coefficients [30]. While the unmodelled unequal probabilities might have disappeared whenever the ‘true’ exposure series were used (and not random permutations as in the ‘unaltered events, random exposures’-scenario), allowing for overdispersion will provide a more robust variance estimator and more valid inference.

The Berkson error associated with aggregating only by date (national analysis) was small in terms of reduced statistical power, but the national analysis came with a higher vulnerability to confounding and overdispersion. So even when it is possible to obtain a large area average exposure that perfectly represents the average of the individual exposures (which will be nearly impossible in real-world examples) aggregating over multiple smaller areas has advantages.

Fung *et al*., investigated the precision of estimates obtained with a case-crossover design and those obtained with a time series design and concluded that, with their models, the latter offered higher precision [31]. This, however, is not a general conclusion; the precision of estimates is determined by several factors. In the ‘events modelled by exposure values’-scenario we obtained a higher proportion of significant coefficients with SY then with SM RSS. This was the result of the higher number of referents per risk, the variance of an estimator with *m* controls is proportional to *m*/(*m* + 1) [8] and the lower correlation of the referents' exposure values.

With respect to the number of referents, we already made two observations: more referents allowed for more power (given that the autocorrelation between referents was limited), but as more referents implied larger strata, exchangeability in matched sets was less likely. In addition, it is important to note that the assumption necessary for unbiased estimation with one to many matchings is different from one to one matching. With one to one matching, pairwise exchangeability becomes a sufficient condition for unbiased estimation. Pairwise exchangeability is a less strong assumption than global exchangeability as exchangeability is only necessary between each risk and each referent separately [25]. Finally, the selection of referents should be without replacement. Levy *et al*. reported an increase in bias as the number of referents increased when sampling with replacement [8].

As previously discussed, conditional Poisson regression allowed for modelling overdispersion through quasi-Poisson regression. One additional advantage of conditional Poisson regression concerns ‘ties’. In a case-crossover study with shared exposures, the exposure values while being continuous in nature, are discretised and can be equal among several of the matched risk-referents sets. When conditioning on event dates instead of unique event identifiers, ties can occur. If ties are present, the likelihood function should take these into account [1]. When the number of ties is large the likelihood quickly becomes incomputable [32].

### Limitations

We only looked into a very limited number of RSS. Full-stratum, semi-symmetric, unidirectional RSS are not investigated in this study. We also did not compare the case-crossover design to alternative designs such as Poisson time series or longitudinal/mixed models.

We used the date of diagnosis as the event date. Preferably we would have used the date of disease onset as the period between infection and diagnosis was likely variable. While this would be problematic if we were to actually analyse the dataset, we did not consider it problematic in this simulation study. The purpose of the events time series was to provide us with a seasonal trend and a long-term trend as they would appear in a real example. In addition, we wanted to investigate overdispersion. This was also possible with our event time series.

While we presented unidentified outbreaks as a possible source of overdispersion and recommended the removal of outlier-dates from the analysis, other aspects of outbreaks are not discussed. In addition, several aspects of infectious diseases such as person-to-person transmission were not investigated.

### Conclusion on case-crossover design

Fixed bidirectional RSS are systematically biased, while time-stratified RSS are not. This bias can, however, be minimised by keeping the proportion of affected matched sets small. All RSS are vulnerable to confounding bias. Researchers looking to detect time-varying confounding could take an approach similar to this simulation study: (1) creating an event time series with the time-varying trends that should be eliminated (both a long-term trend and seasonality in this study), (2) adding causal effects from the exposure time series under investigation and (3) analysing the simulated events and unaltered exposure time series with the desired case-crossover design. The estimated coefficients can then be checked for a confounding bias (if time-stratified RSS were used) or a confounding bias plus a systematic bias (if fixed bidirectional RSS were used). While the latter is a combination of biases, it is not necessarily larger than the former. In addition, the statistical power associated with different RSS can be explored. To illustrate our approach as a tool to compare different RSS, we have investigated an additional time-stratified RSS with 3-week strata and summarised the results in a Supplementary file.

We suggested and demonstrated possible solutions to confounding bias in time-stratified RSS: within matched set modelling and aggregating over smaller areas. Finally, an unexpected high number of events on the same date can inflate type I error and should preferably be removed from the dataset before model fitting.

## Supplementary material

For supplementary material accompanying this paper visit http://dx.doi.org/10.1017/S0950268820000916. click here to view supplementary material

## Availability of data and code

The R code for the simulations and the analysis were provided as Supplementary file. The individual case data and data on the meteorological factors cannot be provided due to privacy concerns.

## Attachment

### Case definitions

Both confirmed and probable cases of *Legionella* spp. were included in the analysis. The laboratory criteria for case confirmation were; (1) the isolation of *Legionella* spp. from respiratory secretions or any normally sterile site [33], (2) detection of *Legionella pneumophila* antigen in urine, (3) a significant rise in specific antibody level to *Legionella pneumophila* serogroup 1 in paired serum samples. The laboratory criteria for a probable case were; (1) detection of *Legionella pneumophila* antigen in respiratory secretions or lung tissue e.g. by DFA staining using monoclonal-antibody derived reagents, (2) detection of *Legionella* spp. nucleic acid in respiratory secretions, lung tissue or any normally sterile site [34, 35], (3) significant rise in specific antibody level to *Legionella pneumophila* other than serogroup 1 or other *Legionella* spp. in paired serum samples, single high level of specific antibody to *Legionella pneumophila* serogroup 1 in serum.

### Description of the data sources

#### National reference centres (NRC)

The NRC analysed isolates they collected themselves and isolates they received from Belgian laboratories. Isolates were sent to the NRC on a voluntary but recommended basis. The goals of an NRC were: confirmation and additional strain characterisation (sero- and genotyping) and determining antibiotic resistance [30]. The NRC for *Legionella* were: ‘Universitair Ziekenhuis Brussel, Vrije Universiteit Brussel’ and ‘Laboratoire Hospitalier Universitaire de Bruxelles (LHUB-ULB), Hôpital Erasme Brussels’.

#### Laboratory sentinel surveillance network

The surveillance started in 1983 and consisted of both hospital laboratories and private laboratories. The network was coordinated by the Institute of Public Health. Participation in the network is voluntary. The coverage of the laboratory sentinel surveillance has been estimated to be around 50% for other pathogens under surveillance, but a specific estimate is unknown for Legionnaires' disease.

#### Mandatory notification

Notification of confirmed Legionnaires' disease cases was mandatory in all three Belgian regions. The notification was coordinated by the regional public health agencies. Physicians and laboratories were obliged to notify cases, but the notification was suspected to be incomplete.
