# Supplementary material for: Optimising the case-crossover design for use in shared exposure settings
Source: Epidemiol Infect. 2020 May 4;148:e151. doi: 10.1017/S0950268820000916 (PMC7374809; doi:10.1017/S0950268820000916)
Supplement: Supplementary file 1 [file S0950268820000916sup001.docx]

Epidemiology and Infection

Optimizing the case-crossover design for use in shared exposure settings

T. Braeye, N. Hens

Supplementary Material

To illustrate our simulation study as a tool to asses confounding associated with referent selection strategies, we introduced a new selection strategy: ‘time-stratified referent selection with three-week strata’ (S3W). The study period was divided into different blocks of three weeks (strata) and the corresponding weekdays within the same strata were selected as referents. For each risk, two referents were selected. This selection strategy has been applied previously in research on the association between legionnaires’ disease and meteorological variables (1–3).

As this is a time-stratified referent selection strategy, we did not expect a systematic bias. In the ‘random dates, unaltered exposures’-scenario we found that this was indeed the case (Supplementary figure S1). Coefficients were estimated at 0 and the proportion of significant coefficients were close to the nominal level (Supplementary figure S2). Because of fewer referents selected for each risk as compared to the other time-stratified referent selection strategies (with S3W two referents for each risk, with SM four/five referents for each risk, with SY 12 referents for each risk), the statistical power associated with S3W will be lower. (The spread in the boxplot for S3W is wider than in SM and SY).


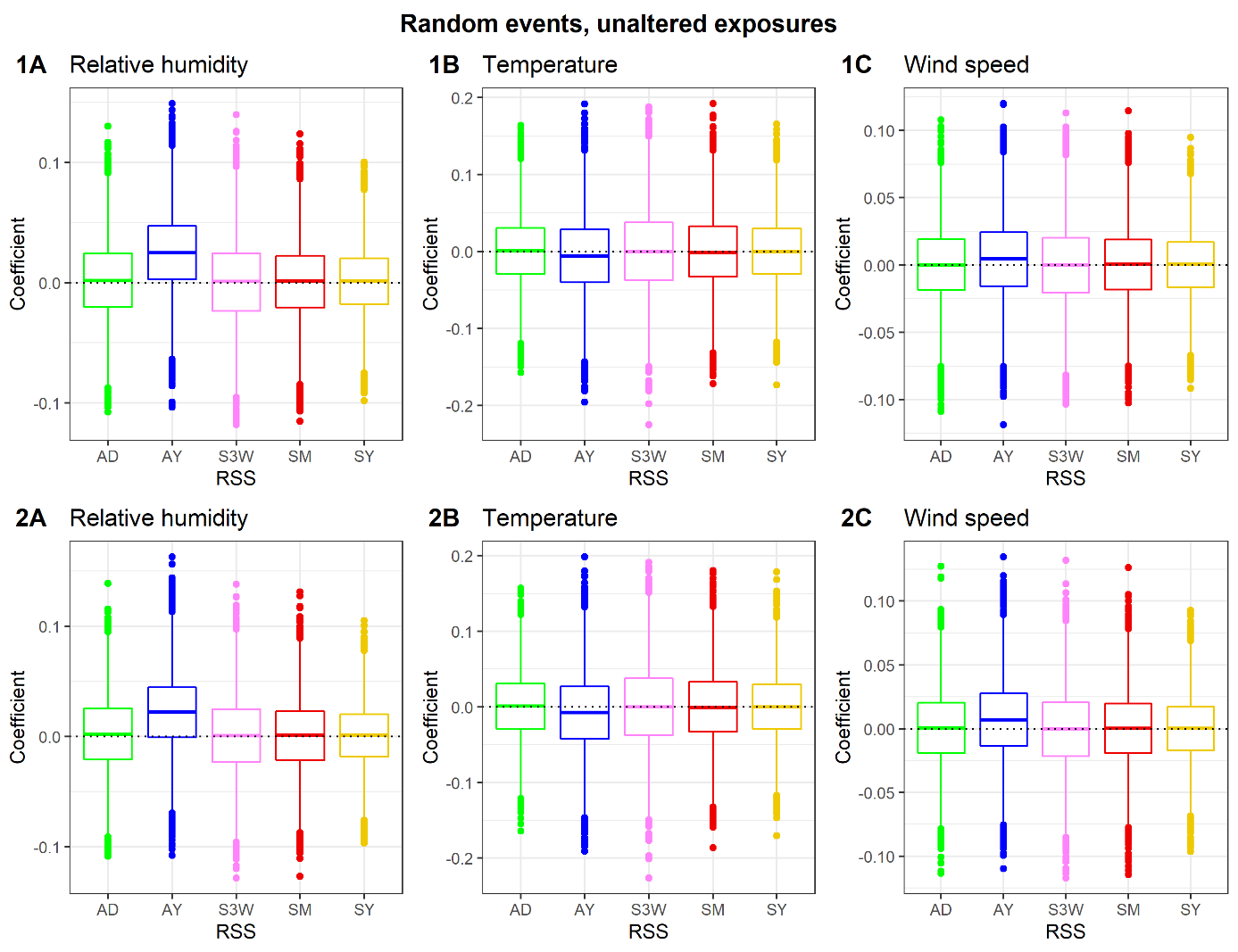


Supplementary figure S1: The ‘random events, unaltered exposures’-scenario. Coefficient estimates for relative humidity (A), temperature (B) and wind speed (C), national (1) and provincial (2) analysis by RSS (AD=Adjacent Days, AY=Adjacent Years, S3W=Strata of three weeks, SM=Strata Month-weekday, SY=Strata Day-of-the-Year).


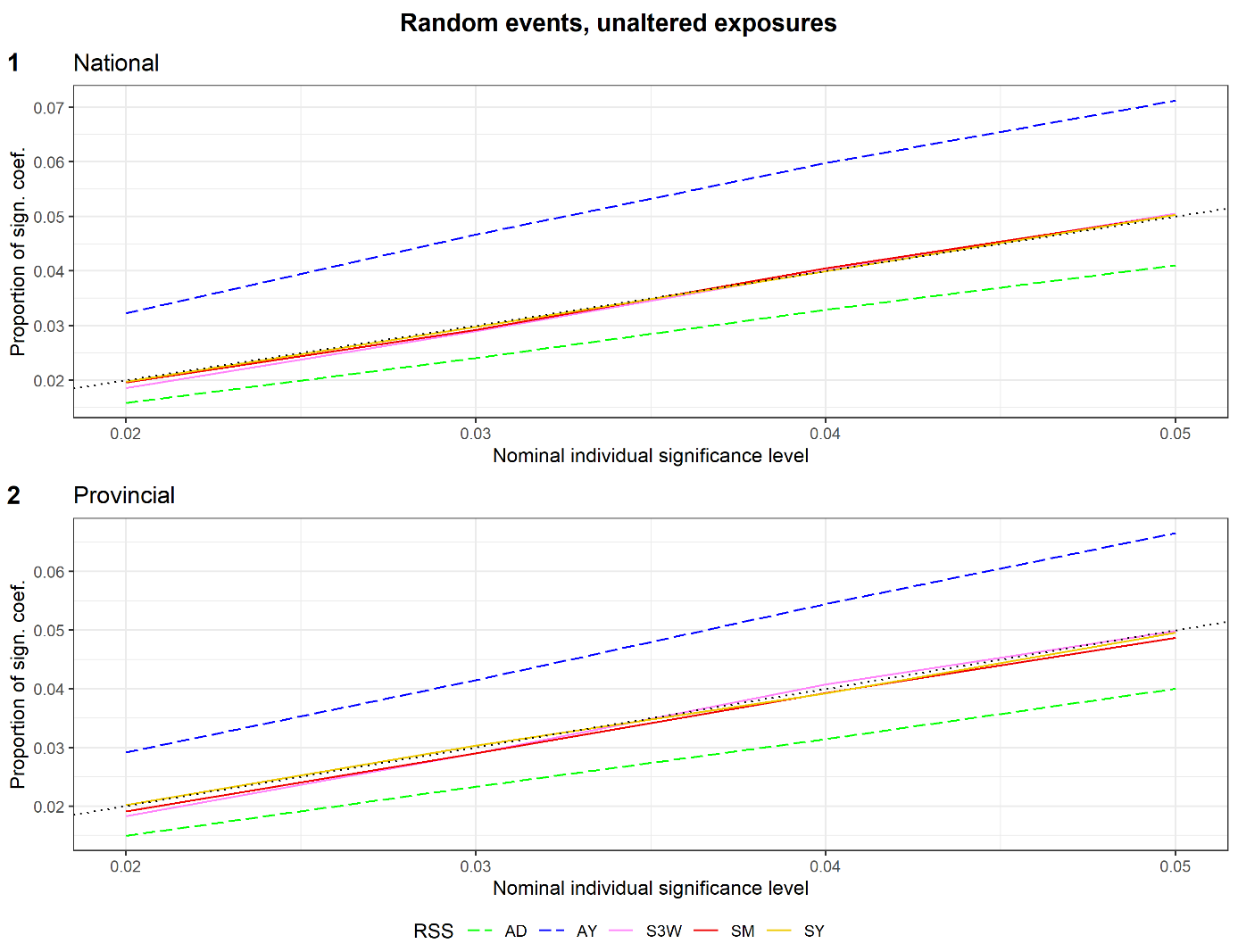


Supplementary figure 2: The ‘random events, unaltered exposures’-scenario. Proportion of significant coefficients over the nominal level for the national (1) and provincial (2) analysis by RSS (AD=Adjacent Days, AY=Adjacent Years, S3W=Strata of three weeks, SM=Strata Month-weekday, SY=Strata Day-of-the-Year).

After the removal of outlier-dates, the S3W selection strategy was associated with a proportion of significant coefficient closer to the nominal level than SM or SY (Supplementary figure S3). This was because less seasonality remained in matched sets of risks and referents with S3W as the strata were shorter (compared to SM) and S3W was much less vulnerable to long-term trends (compared to SY, as S3W spanned a three week period, while SY spanned a 13-year period).


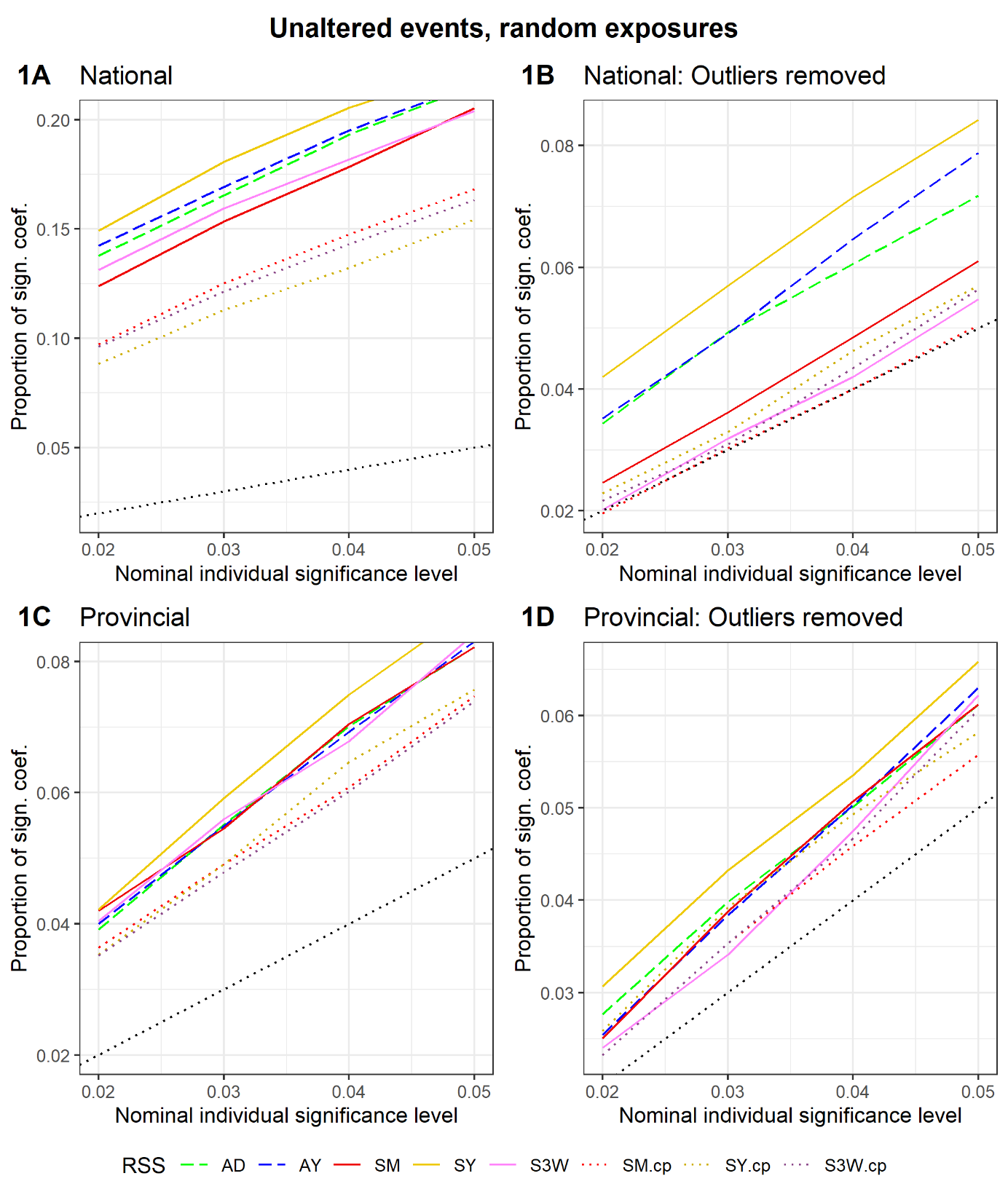


Supplementary figure 3: The ‘Unaltered events, random exposures’-scenario. Proportion of significant coefficients over the nominal level for the national (1) and provincial (2) analysis by RSS (AD=Adjacent Days, AY=Adjacent Years, SM=Strata Month-weekday, SY=Strata Day-of-the-Year, S3W=Strata of three weeks, .cp=conditional quasi-Poisson model).

Finally, there was still seasonal confounding with S3W, the seasonal-trend confounded with trends in the temperature time series (Supplementary figure S4). The confounding bias was smaller if data were aggregated by date and province (provincial analysis) as compared to an analysis after aggregation on date (national analysis).


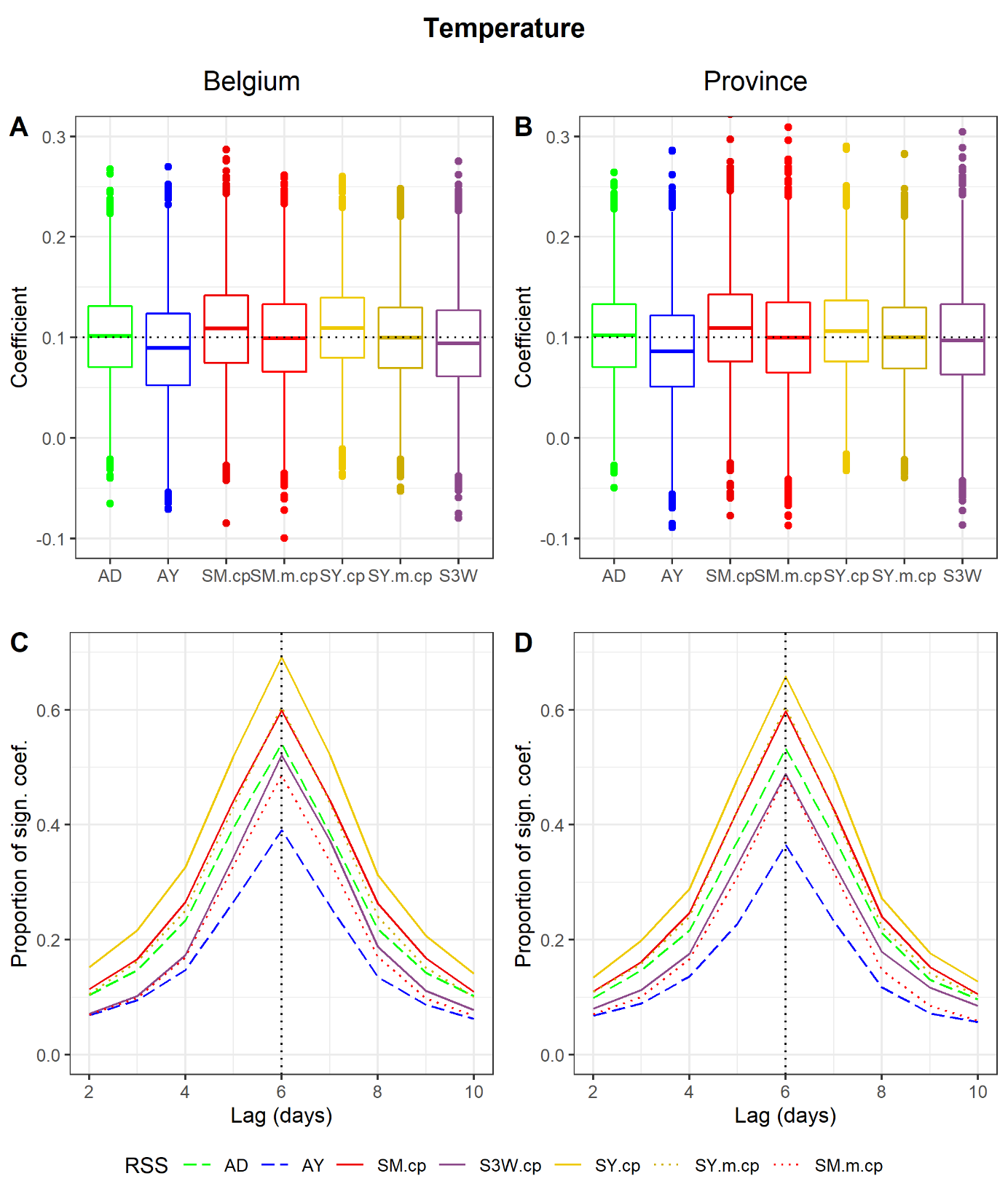


Supplementary figure 4: The ‘event probabilities modelled by exposure values’ scenario. Coefficient estimates for temperature (A, B) and the proportion of significant coefficients over the lag period (in days) (C,D) for the national (A,C) and provincial (B,D) analysis by RSS (AD=Adjacent Days, AY=Adjacent Years, SM=Strata Month-weekday, SY=Strata Day-of-the-Year, S3W=Strata of three weeks, .m=model includes an additional time-varying term, .cp=conditional quasi-Poisson model)

The S3W referent selection strategy thus still resulted in biased coefficients due to seasonal confounding, despite using short 3-week strata. This illustrated that even short strata were vulnerable to seasonal confounding with our exposure time series and that within strata modelling remained necessary with S3W.

1. Gleason JA, Kratz NR, Greeley RD, Fagliano JA. Under the Weather: Legionellosis and Meteorological Factors. EcoHealth. 2016;13(2):293–302.

2. Fisman DN, Lim S, Wellenius GA, Johnson C, Britz P, Gaskins M, et al. It’s Not the Heat, It’s the Humidity: Wet Weather Increases Legionellosis Risk in the Greater Philadelphia Metropolitan Area. J Infect Dis. 2005 Dec 15;192(12):2066–73.

3. Ng V, Tang P, Jamieson F, Drews SJ, Brown S, Low DE, et al. Going with the flow: legionellosis risk in Toronto, Canada is strongly associated with local watershed hydrology. EcoHealth. 2008 Dec;5(4):482–90.
